# Seroprevalence and risk factors of *Toxoplasma gondii* infection in pregnant women following antenatal care at Mizan Aman General Hospital, Bench Maji Zone (BMZ), Ethiopia

**DOI:** 10.1186/s12879-016-1806-6

**Published:** 2016-09-01

**Authors:** Fira Abamecha, Hasen Awel

**Affiliations:** 1 Department of nursing, College of Health Sciences, Mizan-Tepi University, P.O.Box: 260, Mizan-Aman, Ethiopia; 2 Department of Animal science, College of Agriculture and Natural resources, Mizan-Tepi University, P.O.Box: 260, Mizan-Aman, Ethiopia; 3 Department of Health Education and Behavioral Sciences, College of Public Health and Medical Sciences, Jimma University, P.O.Box: 378, Jimma, Ethiopia

**Keywords:** Sero-prevalence, Pregnant women, *T. gondii* infection, Ethiopia

## Abstract

**Background:**

The intracellular parasite, Toxoplasma gondii (*T.gondii*) is found worldwide. Infection with T. gondii during pregnancy can result in fetal and neonatal death or various congenital defects. A serological survey during pregnancy represents a valuable tool for the effective diagnosis and treatment of infected neonates. The aim of this study was to assess the sero-prevalence and risk factors of T.gondii in pregnant women following ante natal care (ANC) services at Mizan Aman General Hospital, Bench Maji zone (BMZ), Ethiopia.

**Methods:**

An institution based cross-sectional study was conducted enrolling a sample of 232 pregnant women attending antenatal care at Mizan Aman General Hospital during 01 December, 2014 to 18 February, 2015. Systematic random sampling technique was used to obtain the required sample. About 5 ml of blood sample was collected aseptically by using properly labeled plain tube with the necessary information. The blood samples centrifuged at 3000 rpm for 10 min to separate serum. The serum was stored at a temperature of 20 °C below zero until the serological analysis was done for the presence of anti T.gondii antibodies (i.e. Immune globulin ‘M’ (IgM) and Immune globulin ‘G’ (IgG)) using enzyme linked immunosorbent assay (ELISA). Exit interview was conducted with eligible mothers to obtain socio-demographic and behavioral data using structured questionnaires. Multivariate logistic regression modeling was employed to identify the potential predictor variables for T.gondii infection. *P*-value less than 5 % was considered to declare a sound significant association.

**Results:**

The response rate of the study was 100 %. The overall sero-prevalence for T.gondii infection was 85.3 % (198/232). About 191 (82.3 %) of the pregnant women were reactive only for IgG anti-bodies. While about 7 (3.0 %) of them were seropositive for both IgG and IgM anti-bodies. None of the mothers were positive for IgM anti-bodies exclusively. On multivariate logistic regression analysis, contact with cat and gardening soil were significantly associated with T.gondii infection (AOR =2.37, 95 % CI = [1.16, 3.57] and AOR = 2.49, 95 % CI = [1.53, 3.86] respectively.

**Conclusions:**

Sero-prevalence of T. gondii antibodies for IgM was relatively high among pregnant women. Contact with cat and soil were risk factors for T.gondii case. Creating awareness on the source of infection, modes of transmission and prevention of T. gondii should be given for pregnant women. Routine screening services for T. gondii infection should be integrated with other ANC services to identify potential infections of the parasite.

## Background

The intracellular parasite*; Toxoplasma gondii* is found worldwide and it is an exceptionally broad host range protozoan parasites on earth. Felines are the only definitive host while all other warm-blooded animals including humans are intermediate hosts for the parasite [1–3]. Toxoplasma infects up to one third of the world’s population, and the infection can be life threatening during pregnancy and in immune-compromised individuals [4].

Humans get infections with *T. gondii* through ingesting of raw or undercooked meat, drinking unpasteurized milk, ingesting of contaminated soil; food or water with cat-shed oocysts, or congenitally via transplacental transmission of tachyzoites [2, 5–7]. Beef and lambs are known to be the most common sources of food related *T.gondii* infections [2]. Needle-stick injuries or cuts, blood transfusion and organ transplantation are also possible risk factors for infection [4].

It is a major public health concern resulting in hospitalizations and this is ranked third in USA among food related causes of death [4, 8, 9]. Infection with *T. gondii* during pregnancy can result in fetal and neonatal death or various congenital defects [5, 10]. Most infected fetuses are likely to have manifestations such as retinochoroiditis, mental retardation, blindness, pneumonias and encephalitis later in their life [11–15].

In Ethiopia, human toxoplasmosis infection is a neglected disease [16] and report of few studies showed that its sero-prevalence in general population ranges from 20.2 % [17] to 90 % [18]. According to Ethiopian Demographic and health survey (EDHS) of 2011, the infant mortality rate in Ethiopia was estimated to be 59 deaths per 1000 live birth [19] and 26 % of them are due to infection [20]. For such occurrences, *T. gondii* might have a great contribution as it has the ability to cause fetal death, spontaneous abortion, still birth, intrauterine growth retardation, preterm deliveries, fetal abnormalities and ocular damage [21, 12].

Maternal *T. gondii* infection is usually asymptomatic and if the diagnosis is delayed, unavoidable and irreversible fetal damage might take place [22, 23]. Therefore, early diagnosis during pregnancy is highly desirable allowing prompt intervention in order to reduce the probability of foetal infections and consequent substantial damages [24]. However, there was no documented data on the sero-prevalence and risk factors of *T. gondii* infection in the study area to the best of our knowledge and only a few studies have been carried out elsewhere in Ethiopia, but not enough to provide basic information that could be used to develop a comprehensive control strategy for the prevention and treatment of *T.gondii* infection. The aim of this study was, therefore, to determine the Seroprevalence and risk factors of *T. gondii* in pregnant women following ante natal care (ANC) at Mizan Aman General Hospital, Bench Maji zone (BMZ), Ethiopia.

## Methods

### Study area and period

The study was conducted in Bench Maji zone (BMZ) which is located in Southern Nations, Nationalities and Peoples Region (SNNPR) and found at distance of 555 km from Addis Ababa (the capital city of Ethiopia). The study was carried out from 01 December, 2014 to 18 February, 2015. The area has appropriate weather conditions conducive to the continued existence of the parasites (i.e. the area is located in ever green zone with annual average temperature and rainfall ranging from 15.1 °C to 27.5 °C and 400 to 2,000 mm respectively, according to BMZ annual report of 2012 and there were large populations of wild and domestic cats, as the report indicated). On top of this, there was no serological screening of pregnant women for *T. gondii* infection in the Hospital in particular and Ethiopia in general.

### Study design and population

An institution based cross-sectional study was conducted at Mizan Aman General Hospital enrolling a sample of 232 pregnant women following ANC services. Sample size was determined using single population proportion formula with sero-prevalence value (*p* = 83.6 %) taken from previous study [25] and 95 % confidence interval with a 5 % desired absolute precision was considered [26]. Considering 10 % of the sample size for non-response rate, the total sample size was calculated to be 232. Two hundred thirty two pregnant women were drawn using a systematic random sampling technique. According to the data obtained from Mizan Aman General Hospital, an average recorded flow rate of pregnant women for ANC in the preceding year was 15 women per day. Our study period was estimated to be 78 days (i.e. form 01 December 2014 to 18 February 2015) and therefore about, 1170 (15*78) pregnant women are expected to attend ANC during the study period. This gives a sampling interval (K^th^ value) of five (i.e. 1170 divided by 232 = 5). Finally, a sample unit (a participant) was selected from every five records picking one random record at once.

### Questionnaire survey

Exit interview was done to collect data about the potential risk factors by using structured questionnaire. The questionnaire covers socio-demographic information including age, level of education, occupation, residence, source of drinking water, and obstetric history and other behavioral factors like consumption of raw vegetables, raw milk and raw meat, and hand washing practices. Furthermore, presence of wild and domestic cats in their home, neighboring or surroundings, number of cats in the home, contact with cats and soil were addressed.

### Sample collection and serological analysis

We used a blood sample collected for other routine purposes in the hospital care. About 5 ml of venous blood specimen were collected aseptically by using plain tube. The tubes were labeled properly with the necessary information. Then, the whole blood samples were left for few hours at room temperature to allow clotting, and then centrifuged at 3000 rpm for 10 min to separate serum. The serum was stored at 20 °C below zero until it was analyzed serologically for anti-Toxoplasma antibodies using the enzyme linked immunosorbent assay (ELISA) test. Commercial kit from (*HUMAN Gesellschaft für Biochemica und Diagnostica mbH, Germany*); was used to measure *T. gondii* IgG and IgM antibodies and the test was performed according to the manufacturer’s instructions. The cut-off value was expressed in an index. The test was considered negative if the index was <0.77 and positive if it was >0.97 for IgM. In the same way, the cut-off values for detection of IgG <0.3 was negative and ≥0.6 was considered positive.

### Data analysis

Data were coded and entered into Epidata 3.1 statistical packages (Jens M. Lauritsen and Michael Bruus: EpiData Association, Denmark). The data were imported to statistical package for social sciences (SPSS) version 20 for windows (v 20.0; IBM Corporation, Armonk, NY, USA) for further analysis. Cross-tabulations of sero-status were done with socio-demographic and behavioral characteristic as summary measures. Univariate logistic regression was employed as bivariate analysis to select significant variables to be used in subsequent multivariate logistic regression analysis. Multivariate logistic regression analysis was used to calculate adjusted odds ratios (AOR), with variables resulting in *p*-values less than 0.05 considered to be significantly associated with seropositivity.

## Results

### Sero-positivity of *T.gondii*

A total of 232 pregnant women were enrolled during the study period with the mean age ± standard deviation (SD) of 23.65 ± 5.4. An overall sero-prevalence for T.gondii infection was 85.3 % (198/232) [95 % CI: 80.1, 89.4]. About 191 (82.3 %) of the pregnant women were reactive only for IgG anti-bodies. While only, 7 (3.0 %) of them were seropositive for both IgG and IgM anti-bodies. None of the mothers were positive for IgM anti-bodies exclusively. Of the 198 seropositive pregnant women, 100 (50.5 %) were in the second trimester and 22 (11.1 %) were with history of abortion.

Fifty six, (28.3 %) and 87 (43.9 %) women with seropositivity had reported the presence of either one or more cats in their house and their neighbors respectively. One hundred and twenty one, (61.1 %) women had contact with cats. Ninety three, 47 % of respondents consumed raw meat with 92 (46.7 %) having contact with gardening soil. Majority of them, 156 (78.8 %) consumed boiled milk while 177 (89.4) wash vegetable before consuming, and 163 (82.3) were tap water for drinking. (Tables 1 and 2).

**Table 1:** General characteristics of the pregnant women Attending ANC at Mizan-Aman General hospital, south west Ethiopia, 2015

Variable	IgG Positive (*n* = 198)	Percent	*P* value
Age category			
16–20	64	32.3	0.011
21–30	124	62.6	
> 30	10	5.1	
Occupation			
government employee	35	17.7	0.886
Housewife	129	65.2	
Other	34	17.2	
Residence			
Urban	135	68.2	0.333
Rural	63	32.8	
Education level			
Can’t read and write	48	24.2	0.033
Can only Read and write	32	16.2	
Primary	55	27.8	
Secondary	35	17.7	
Tertiary	28	14.1	
Number of Gravida			
One	77	38.9	0.223
More than one	121	61.1	
Stage of pregnancy			
First trimester	56	28.3	0.346
Second trimester	100	50.5	
Third trimester	42	21.2	
History of abortion			
Yes	22	11.1	0.395
No	99	50.0	
Not applicable	77	38.9

**Table 2:** Seropositivity of *T. gondii* in relation to behavioral characteristics of the pregnant women in Mizan-Aman General hospital, south west Ethiopia

Variable	IgG Positive (*n* = 198)	Percent	*P* value
Ownership of cat			
Yes	56	28.3	0.016
No	142	72.7	
Presence of cat in the neighborhood			
Yes	87	43.9	0.330
No	111	56.1	
Having close contact with cat			
Yes	121	61.1	0.002
No	77	38.9	
Consumption of raw meat			
Yes	93	47.0	0.011
No	105	53.0	
Consumption of boiled milk			
No	42	21.2	0.761
Yes	156	78.8	
Hand washing after contact with raw meat			
Yes	177	89.4	0.395
No	21	10.6	
Having contact with garden soil			
Yes	92	46.5	0.001
No	106	53.5	
Consuming Vegetable without washing			
Yes	21	10.6	0.840
No	177	89.4	
Source of drinking Water			
Tap	163	82.3	0.642
Well	29	14.7	
Others	5	2.5	

### Risk factors to *T.gondii* infection

The result of univariate logistic regression analysis showed that consumption of raw milk, hand washing practices, consumption of unwashed vegetables, sources of drinking water were not significantly associated with seropositivity, while women’s age, presence of cat in the neighborhood, consumption of raw meat, contact with cats and contact with soil were significantly associated with seropositivity and, thus, were included in multivariate analysis. Finally, the result of multivariate logistic regression analysis showed that women’s contact with cats and gardening soil were potential risk factors of *T. gondii* infection with OR = 2.37 and 95 % CI [1.16, 3.57] and OR = 2.49 and 95 % CI = [1.53, 3.86] respectively (Table 3).

**Table 3:** Independent Predictors of *T. gondii S*eroprevalence among pregnant women attending ANC at Mizan-Aman General hospital, south west Ethiopia, 2015

Variable	IgG Positive (*n*)	COR (95 % CI)	AOR (95 % CI)	*P*-value
Residence				
Urban	135	Ref.	Ref.	0.421
Rural	63	0.66 [0.28, 1.54]	1.52 [0.64, 3.62]	
Occupation				
Housewives	129	Ref.	Ref.	
Employed	35	0.79 [0.31, 2.01]	0.78 [0.30, 2.02]	0.392
Others	34	0.83 [0.25, 2.73]	0.72 [0.21, 2.43]	0.214
Ownership of cats				
No	142	Ref…	Ref.	
Yes	56	1.4 [0.180, 2.62]	0.89 [0.52, 2.29]	0.065
Contact with cat				
No	77	Ref	Ref.	
Yes	121	2.01 [1.48, 3.64]	2.37 [1.16, 3.57]	0.010*
Consumption of raw meat				
No	105	Ref.	Ref	
Yes	93	2.71 [1.23, 5.96]	2.03 [0.84, 4.93]	0.122
Age category				
16–20	70	Ref.	Ref.	
21–30	103	0.741 [0.31, 1.77]	0.26 [.69, 1.21]	0.242
> 30	25	2.8 [1.08- 7.25]	0.98 [.11, 2.07]	0321
Education level				
Illiterate	48	Ref.	Ref.	
can read and write	40	1.20 [0.43, 3.31]	0.66 [0.15, 2.97]	0.642
primary	36	1.77 [0.68, 4.67]	0.69 [0.18, 2.50]	0.283
Secondary	46	0.23 [0.05, 1.13]	1.67 [0.51, 5.53]	0.261
Tertiary	28	0.38 [0.08, 1.88]	3.3 [0.94, 11.78]	0.083
Contact with soil				
No	106	Ref.	Ref.	
Yes	92	1.32 [1.48, 3.16)	2.49 [1.53, 3.86)	0.002*
Stages of pregnancy				
1^st^ trimester	56	Ref.	Ref.	
2^nd^ trimester	100	0.61 [0.23, 1.62]	0.64 [0.24, 1.69]	0.284
3^rd^ trimester	42	0.54 [0.22, 1.27]	0.56 [0.23, 1.35]	0.453
Number of Gravida				
One	77	Ref.	Ref.	
More than one	121	0.64 [0.31, 1.32]	1.46 [0.69, 3.08]	0.122

*COR* Crude odds ratio, *AOR* adjusted odds ratio, *CI* Confidence interval, *Significant association (*P* < 0.05)

## Discussion

This is among few studies in Ethiopia and the first study conducted among pregnant women in women attending ANC at Mizan-Aman General hospital, Southwest part of the country to determine *T. gondii* infection and its risk factors among this group.

The overall sero-prevalence of *T. gondii* infection in this study was found to be 85.3 % (95 % CI: [80.1, 89.4]. This is in-line with the 86.4 % and 83.6 % of sero-prevalence reported from central and south eastern Ethiopia, respectively [16, 25]. However, it is significantly higher than recent study conducted in Ethiopia, 68.4 % [27] and lower than study done in Ghana, 92.5 % [28]. In contrast, lower sero-prevalence of *T. gondii* was reported in many European countries and the United States of America [29, 30]. The reported sero-prevalence rate in pregnant women varies between countries as well as different areas within the same country [31, 32].

Several factors such as differences in climatic conditions, where higher sero-prevalence is associated with hotter and wetter areas which mainly support sporulation of oocysts compared to less humid areas [33–35] and cat density with high rate of oocyst shedding were reported before [16]. Furthermore, the difference could be due to mothers’ socio-economic characteristics such as management of cats, educational level, hygienic practice, feeding habit and sensitivity difference in the serological tests employed [2].

The 82.3 % Toxoplasma IgG positive and lgM negative results obtained in this study was similar with study from Jimma town, Ethiopia [25] and higher than those studies reported from Central Ethiopia [16] and elsewhere in the world [36–40]. In this study, 3.02 % of enrolled pregnant women had detectable IgM antibodies during pregnancy with potential risk of congenital T. gondii infection warranting attention to design preventive measures. This may happen due to either recrudesce of previous infection because of reduction of body’s resistance related to pregnancy or due to re-exposure to *T. gondii* infection [40].

Approximately half of pregnant women (49.14 %) were in the second trimester of gestational period. Pregnant women are more susceptible due to immunosuppressant condition of pregnancy where the innate immunity protecting against *T. gondii* is more altered during the third trimester of gestation [40].

According to this study, pregnant women having close contact with cats were at risk for *T.gondii* infection showing similar results with studies carried out in Ethiopia [27] and in Taiwan [41, 42]. Evidence that cats are definitive host, where sexual multiplication of *T. gondii* takes place and excrete the unsporulated oocysts with feces had been previously confirmed [43–45]. Pregnant women in contact with infected cats will naturally be at greater risk of acquiring the infection [2, 30]. In contrast, contact with cats was also not associated to the chance of infection as reported from UK, Brazil, Turkey and Nigeria [46–49]. The risk of infection might exist when there is close contact with cats or with their feces that could remain in the environment for at least 24 h so that the oocysts sporulate and become infective [50, 51].

Having contact with soil demonstrated significant association with *Toxoplasma* sero-positivity of pregnant women in this study. Similar result was reported from Central Ethiopia [16]. On the other hand, Ethiopian studies did not find an association between infection and contact with gardening soil [25, 27]. Similarly, study conducted in Brazil [52] did not find an association between *T. gondii* infection and gardening probably because of the climate of the region, which is dry and hot throughout the year [53]. The habit of handling soil or sand should also be considered in *T.gondii* infection. On the other hand, another study done in Southern Brazil [54] showed that contact with the contaminated soil was the most important factor. The oocysts in soil can remain infective for 12 to 24 months under ideal moisture, temperature conditions and in a favorable shady location [51].

Differences in sero-prevalence for *T. gondii* infections of this study were not statistically significant for educational status. This finding was different from a study done in Debre Tabor town, Ethiopia [27], which reported pregnant women with low educational status had higher prevalence of *T.gondii* antibody than with higher educational status. Similar results were reported from Brazil [40] and China [55] that noticed maternal school education presented a clear protecting effect for *anti-T.gondii* sero-positivity. High education level may reduce risk of exposure and increase awareness to adopt appropriate hygienic measures [56].

One of the risk factors that are often associated with acute infection in pregnant women was eating raw or undercooked meat [56]. Ethiopia has a long history of eating of raw meat locally known as *‘Kurt’* in Amharic language [16], which is still popular. In the present study, there was no significant association between consumption of raw meat and *Toxoplasma* infection. This is different from earlier studies in Ethiopia [57, 58] and elsewhere [2, 38, 48] that demonstrated significant association between sero-positivity and behavior of raw meat consumption. However, report of Debre Tabor and Jimma town, Ethiopia [27] and [25] respectively, did not find significant association of sero-prevalence of *T.gondii* with raw meat consumption. This difference could be due to frequency of consumption, type of consumed meat (pig, sheep and goat) and prevalence of the parasite in the animals [2, 59].

## Conclusion

To sum, sero-prevalence of *T. gondii* antibodies was relatively high among pregnant women. In this study, having contact with cat and gardening soil were found to be independent predictors of *T. gondii* infection. Results of the present study therefore, advocate implementation of preventive measures. Creating awareness on the source of infection, modes of transmission and prevention of *T. gondii* should be given for pregnant women. Routine screening services for *T. gondii* infection should be integrated with other ANC services to identify potential infections of the parasite.

## Abbreviation

ANC: Ante natal care
AOR: Adjusted odds ratio
EDHS: Ethiopian health and demographic survey
ELISA: Enzyme linked immunosorbent assay
IgG: Immuno globulin G
IgM: Immunoglobulin M
